# Variability of endocrine findings with age and clinical severity in the long-term follow-up of pseudohypoparathyroidism: evidence of TRH resistance with central hypothyroidism

**DOI:** 10.3389/fendo.2026.1891226

**Published:** 2026-07-27

**Authors:** Burcu Senkalfa, Yagmur Unsal, Nur Berna Celik Ertas, Zeynep Alev Ozon, Dogus Vurallı, Murat Bastepe, Elmas Nazlı Gonc

**Affiliations:** 1 Division of Pediatric Endocrinology, Department of Pediatrics, Faculty of Medicine, Hacettepe University, Ankara, Türkiye; 2 Endocrine Unit, Department of Medicine, Massachusetts General Hospital and Harvard Medical School, Boston, MA, United States

**Keywords:** endocrine findings, genotype phenotype correlation, natural course, pseudohypoparathyreoidism, TRH resistance

## Abstract

**Background:**

Pseudohypoparathyroidism (PHP) is a rare disorder with clinical and genetic heterogeneity, resulting from inactivating variants or epigenetic alterations of the *GNAS* locus.

**Objective:**

To characterize the natural course of PHP, focusing on rarely defined endocrine features.

**Methods:**

Nineteen children (11 girls) from 17 families from a single center, diagnosed with PHP from 1992 to 2025, were enrolled. Genetic/epigenetic analyses, age at onset, and evidence of hormone resistance, radiologic findings, and long-term follow-up of anthropometric measurements were retrospectively reviewed, and cases were classified according to clinical and genetic features as PHP1A, PHP1B, or PPHP.

**Results:**

The study group consisted of children with PHP1A (n=10), PHP1B (n=8), and PPHP (n=1). PHP1A cases harbored six different *GNAS* point mutations, one of which was novel. PHP1Bs exhibited *GNAS* methylation abnormalities. Except for one PHP1A case, all PHP1A/1Bs displayed PTH resistance, which developed earlier in PHP1A (6 years vs 11.2 years). All PHP1A and 5 PHP1B cases had clinical or subclinical hypothyroidism. One PHP1A and two PHP1B cases had low free T4 with inappropriately normal TSH, suggesting central hypothyroidism due to presumed TRH resistance. Clinical signs of gonadal dysfunction were observed in four of six girls with PHP1A. Skeletal features of AHO were present in 7 PHP1A and 3 PHP1B patients. Intellectual disability was identified in 50% of PHP1A. Phenotypic variability was noted among subjects with the same mutation.

**Conclusion:**

Overlapping PHP1A and PHP1B features may complicate differential diagnosis, necessitating molecular genetic analysis. The pattern and severity of endocrine involvement, including possible central hypothyroidism, vary widely among affected children.

## Introduction

Pseudohypoparathyroidism (PHP), initially described by Fuller Albright and colleagues, is a disorder characterized by resistance to parathyroid hormone (PTH). PHP has been classified into type I and type II according to the nature of nephrogenous response to PTH administration (1, 2). PHP type I, where PTH-induced elevation of both urinary cAMP and phosphate is blunted, results from defects in the imprinted *GNAS* that diminish the expression or the activity of its major product, the stimulatory G protein alpha-subunit (Gsα) (1, 2). PHP type I is further subdivided into several subtypes based on molecular mechanisms, inheritance patterns, and DNA methylation abnormalities: PHP type 1A (PHP1A), PHP type 1B (PHP1B), and pseudopseudohypoparathyroidism (PPHP) (3). Gsα is a signaling protein that mediates the actions of numerous endogenous ligands via cAMP generation. While it is biallelically expressed in many tissues, the paternal Gsα allele is silenced in a number of tissues including the proximal renal tubules, pituitary, thyroid gland, and certain parts of the hypothalamus, making the maternal allele the predominant source of Gsα (4, 5). Consequently, genetic or epigenetic defects affecting the maternal *GNAS* allele led to severely impaired Gsα-dependent receptor signaling in these tissues (6).

Caused by heterozygous inactivating mutations within the Gsα-coding exons of the maternal *GNAS* allele, PHP1A is characterized by resistance to multiple hormones, including PTH, TSH, gonadotropins, and GHRH, as well as Albright hereditary osteodystrophy (AHO), which encompasses short stature, obesity, round face, brachydactyly, heterotopic subcutaneous ossifications, and intellectual disability (3). The patients often develop obesity within the first two years of life (7). The severity of cognitive impairment is heterogeneous, with 30% showing normal cognitive development (3, 8). PTH resistance is the most common form of hormone resistance and is usually identified in childhood (7, 9). Evidence of TSH resistance can be detected during the neonatal period. Inactivating variants affecting Gsα-coding exons of the paternal *GNAS* allele cause PPHP, which manifests as AHO phenotype, without hormone resistance (3). Thus, AHO features are present in patients with Gsα-coding mutations regardless of the parental origin. However, studies suggest that obesity and intellectual disability are features that are primarily found in PHP1A rather than PPHP cases (10, 11).

On the other hand, PHP1B, is caused by epigenetic defects (aberrant methylation) of the maternal *GNAS* allele, with all reported cases showing a loss of methylation at the A/B differentially methylated region in the absence or presence of additional methylation abnormalities in this locus (12, 13). The A/B loss of methylation results in the silencing of both Gsα alleles in those tissues in which the paternal Gsα allele is normally silenced, thereby leading to severe Gsα deficiency. The recurrent genetic defect, particularly found in familial cases, is a maternal microdeletion with the neighboring *STX16* gene (14), which disrupts a long-range enhancer of one of *GNAS* transcripts that is crucial for maintaining A/B methylation in the early embryo (15). PHP1B patients present with isolated PTH resistance, with mild TSH resistance manifesting in a substantial number of cases. Typically, the hormone resistance is not accompanied by AHO phenotype, although several cases with mild brachydactyly or other skeletal manifestations have been observed (16).

Clinical features across PHP subtypes regarding both the extent and type of hormone resistance are heterogeneous and variable among reported cases (17, 18). A clear relationship between the type or location of *GNAS* mutation and the timing and severity of hormone resistance or the AHO phenotype has not been established. A collection of additional, well-characterized cohorts is expected to further elucidate the natural history and genotype-phenotype correlation. Therefore, the aim of this study was to describe the natural course of clinical, laboratory, and radiologic characteristics in our PHP patients.

## Materials and method

### Participants

The study was carried out in a tertiary center. 19 cases (F/M: 10/9) from 17 families diagnosed with PHP from 1992 to 2025 were enrolled. Demographic characteristics (sex, age at presentation, and diagnosis), presenting symptoms, anthropometric measurements (body weight, height, body mass index (BMI)), physical examination, pubertal staging and laboratory examination (albumin, calcium, phosphorus, PTH, alkaline phosphatase (ALP), 25-hydroxyvitamin D, TSH, free thyroxine (FT4), follicle-stimulating hormone (FSH), luteinizing hormone (LH), estradiol, total testosterone, and GH stimulation tests), age at obesity onset, each hormone resistance, and results of genetic analysis were retrospectively retrieved from medical records. Follow-up assessments were performed every 3–6 months in the outpatient setting. Participants who have reached adulthood or were lost to follow-up were recalled for a final evaluation. The study was conducted in accordance with the Declaration of Helsinki and was approved by the local ethics committee (Approval number: SBA 25/330). Informed consent was waived due to the retrospective nature of the study.

### Anthropometric measurements

Anthropometric measurements were performed by a nurse trained in auxology. Bodyweight was measured as kilograms (kg) using a digital weighing scale (SECA 769, Hammer Steindamm, 22089, Hamburg, Germany), wearing lightweight clothing with a sensitivity of 0.1 kg. Height was measured in meters (m) with participants in a standing position using a stadiometer (SECA 264, Hammer Steindamm, 22089, Hamburg, Germany) with a sensitivity of 0.1 cm. Bodyweight and height standard deviation scores (SDS) were calculated using gender specific charts with respect to chronological age. Short stature was determined if height-SDS was <-2 SDS according to sex and chronological age-matched charts. BMI was calculated as weight (kg)/height (m²). Then, BMI percentiles were assessed with respect to sex and chronological age. Children with a BMI of 85^th^ to 94^th^ percentile and adults with a BMI of 25–29 kg/m² were defined as overweight. Children with a BMI>95th percentile and adults with a BMI>30 kg/m² were defined as obese. Children with a BMI>99^th^ percentile and adults with a BMI>40 kg/m² were defined as severe obesity.

### Albright hereditary osteodystrophy phenotype

AHO features were considered as short stature, obesity with a round face, short metacarpals, heterotopic ossification with or without intellectual disability (3). Neurocognitive evaluation relied on the history of psychomotor development, academic performance, and/or a standardized psychometric test (Wechsler Intelligence Scale for Children-Revised (WISC-R)) when available and was carried out by a developmental-behavioral pediatrician.

### Diagnosis and classification of PHP

PHP was diagnosed based on clinical, laboratory, radiologic, and molecular genetic findings. Patients displaying hormone resistance due to a heterozygous inactivating mutation in *GNAS*, regardless of the presence of an AHO phenotype, were categorized as PHP1A. Participants with hormone resistance and a methylation defect in *GNAS* were categorized as PTH1B. Each patient’s family members with PHP1A underwent clinical evaluation for findings of PHP. Genetic tests were performed if clinically indicated (Family III).

Case XVII-1 was born at 34 weeks’ gestation with a birth weight of 1700 g (8th percentile). She exhibited an AHO phenotype, including short stature (height −2.8 SDS), short metacarpals, and osteoma cutis. Her mid-parental height was 166 cm (0.4 SDS). She was followed until 13.6 years of age without developing hormone resistance and was therefore classified as PPHP; genetic analysis was not performed.

### TSH and TRH resistance

Elevated serum TSH with normal or slightly decreased FT4 was defined as TSH resistance. Low FT4 with normal TSH concentration was defined as central hypothyroidism due to TRH resistance. Normal values of FT4 and TSH were 7.8-13.7 pmol/L and 0.38- 5.33 mIU/L respectively.

### PTH resistance

PTH resistance was defined as low (serum calcium<8.5 mg/dL) or low-normal serum calcium along with relatively elevated serum PTH (serum PTH: 12–88 pg/mL) without vitamin D deficiency.

### Gonadotropin resistance

Pubertal assessment was conducted by an experienced pediatric endocrinologist. Testicular volume was estimated by palpation to the nearest 1mL using Prader’s orchidometer, and pubertal stage was determined using Marshall and Tanner staging (19, 20). Gonadotropins were measured at pubertal age. Gonadotropin resistance was defined in patients with oligomenorrhea/amenorrhea, delayed puberty, arrested pubertal development, and/or infertility who had elevated gonadotropin levels (FSH and/or LH at or above the upper reference limit) despite low age-appropriate estradiol concentrations in girls or low total testosterone concentrations in boys. If pubertal findings (testicular volume exceeding 4 mL in boys and thelarche in girls) started 2-2.5 standard deviations later than the population mean (14 years in boys and 13 years in girls), it was defined as delayed puberty (21). If puberty began within the normal age range but pubertal progression substantially slowed down or was arrested (i.e., puberty was not completed within five years in boys, within three years in girls, or menarche had not occurred by 15 years of age), it was also classified as delayed puberty (21). Oligomenorrhea was defined as having fewer than nine menstrual periods per year and menstrual cycles longer than 35 days (22).

### Radiologic examination

Postero-anterior left-hand radiography was evaluated by an experienced pediatric endocrinologist. Participants with short 4^th^ and 5^th^ metacarpals and/or Madelung deformity were recorded. Radiologic investigation for subcutaneous ossification was performed only when indicated by clinical findings.

### Genetic analysis

Genomic DNA was extracted from peripheral blood leukocytes using standard protocols. Targeted next-generation sequencing (NGS) of genes involved in *GNAS*-related signaling pathways was performed, with a minimum mean coverage of ≥100× and >98% of target regions covered at ≥20×. Bioinformatic analysis included alignment to the GRCh38, variant calling, and annotation using databases such as ClinVar, gnomAD, and the ClinGen. Functional effects were predicted using Ensembl Variant Effect Predictor (VEP).

Due to the complexity of the *GNAS* locus, all variants were standardized to the MANE Select transcript (NM_000516.7). Variant classification followed the guidelines of the American College of Medical Genetics and Genomics and the Association for Molecular Pathology (19). Truncating variants, including nonsense and frameshift variants, were considered strong evidence of pathogenicity (PVS1), while additional criteria (PM2, PS4, PP4) were applied as appropriate. Missense variants were interpreted cautiously, considering in silico predictions, evolutionary conservation, population frequency, and available clinical data. In the absence of functional validation, segregation data, or consistent evidence in the literature, such variants were classified as variants of uncertain significance (VUS).

In patients without detectable pathogenic variants in *GNAS* coding exons, methylation-specific multiplex ligation-dependent probe amplification (MS-MLPA) was performed using the SALSA MLPA KIT ME031-*GNAS* (MRC-Holland, Amsterdam, the Netherlands) to assess the methylation status of *GNAS* differentially methylated regions (A/B, NESP-AS, XL, and NESP55).

### Statistical analysis

Analysis was conducted using SPSS 23.0 for Windows software package (IBM Corp., Armonk, NY). Normality of the numeric variables was evaluated using the Shapiro-Wilk test (if n<50). Descriptive statistics for categorical variables were presented as frequency (n) and percentage (%), while numeric variables were reported as median, minimum, and maximum. Group comparisons of non-normally distributed continuous variables were performed using the Mann–Whitney U test.

## Results

**Figure 1:**
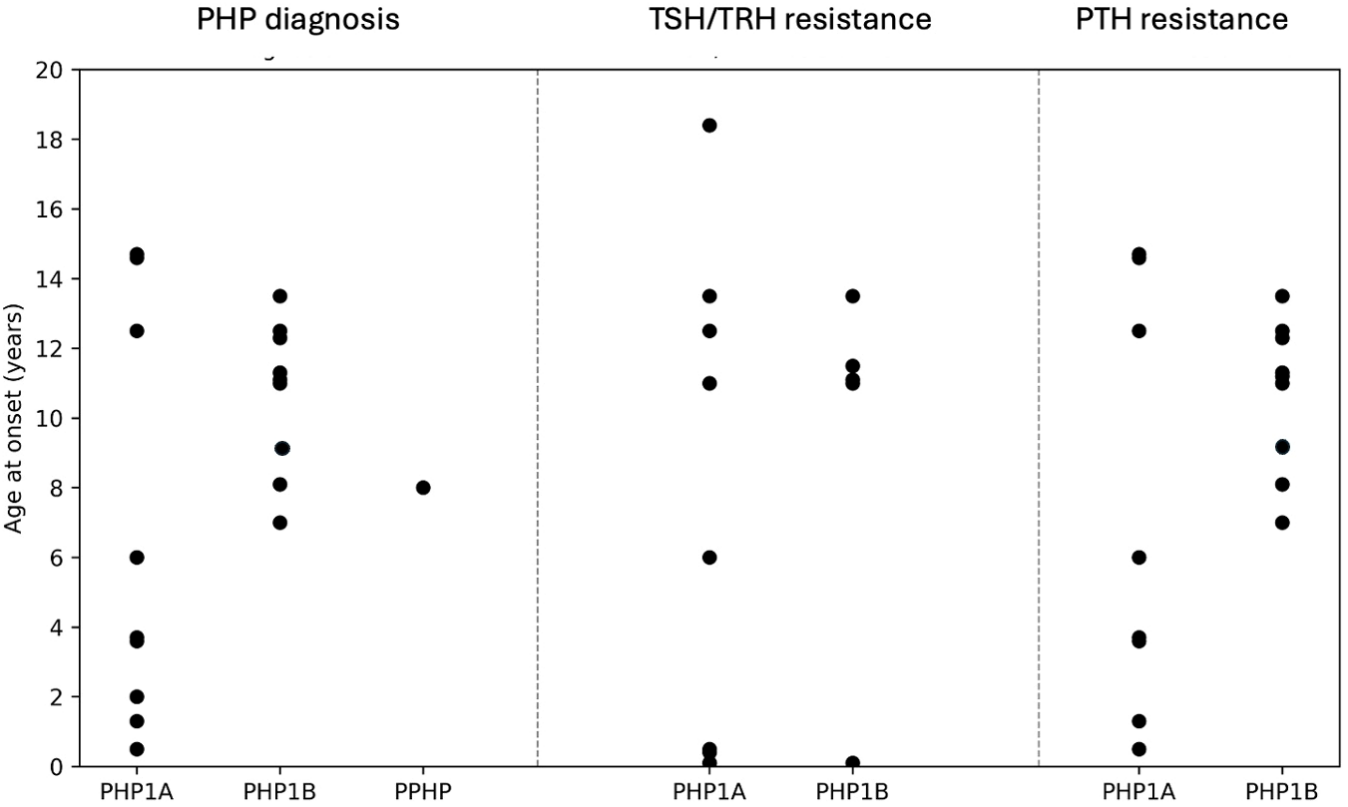
Age distribution at diagnosis of PHP and at the onset of TSH/TRH and PTH resistance in PHP1A, PHP1B and PHPP.

Nineteen cases (11 girls) with PHP from 17 families were included. The median age at presentation was 7 years (0-14.7); it was 3.2 years (0-14.7) for 10 cases (6 girls) with PHP1A, and 10.3 (0-12.5) for 8 cases (4 girls) with PHP1B. Median age at diagnosis of PHP was 8.1 years (0.5-14.7) (**Figure 1**). Case XVII-1 was presented at the age of 5.6 years and diagnosed with PPHP at the age of 8 years (**Figure 1**). Following a median follow-up of 4.2 years (0–33), the median age at the final clinical visit for the study cohort was 5.8 years (4.3-39).

### Genetic analysis

Genetic analysis was performed in 18 cases from 16 different families (10 PHP1A, 8 PHP1B) (**Table 1**). Nine cases from eight families had six different heterozygous *GNAS* point mutations, one of which was novel (case IV), and were diagnosed with PHP1A (**Table 1**). Detection of a variant was noted in the medical records of Case I-1; however, the specific variant was not recorded; thus, phenotypic findings led to a diagnosis of PHP1A. An identical variant was detected in three siblings from Family III. Segregation analysis revealed that their mother also carried the same variant (**Table 1**). Eight cases with PHP1B demonstrated loss of methylation in the *GNAS* complex locus (*NESP55-1, GNAS-AS1, GNASXL*, and *GNAS A/B* (n=1)*, NESPAS, GNASXL*, and *GNAS* A/B loci (n=5), *NESPAS* and *GNAS* A/B loci (n=1), and *GNAS* A/B loci (n=1)) (**Table 1**), none of them exhibited STX16 deletion. Genetic analysis was not performed in Case XVII-1; nevertheless, due to the presence of the AHO phenotype (short stature, short metacarpal bones, and osteoma cutis), and low birth weight without any evidence of hormone resistance until the age of 13.6, a clinical diagnosis of PPHP was established (**Table 1**).

**Table 1:** Genetic, clinical and endocrine features of cases with pseudohypoparathyroidism.

			Genetic evaluation	Clinical findings											Endocrine findings				
				Presentation		Diagnosis			Last clinical visit			Accompanying features			TSH/TRH resistance	PTH resistance	GHD	Gonadotropin resistance	
Family	Case	Sex	*GNAS* variant (NM_000516.7)	Age (year)	Presenting finding	Age (year)	Height SDS	BMI SDS	Age (year)	Height SDS	BMI SDS	Obesity onset (year)	ID	SF	Age^c^ (year)	Age^c^ (year)	Age^c^ (year)	Age^c^ (year)	
Type 1a																			
I	I-1	M	+^a^	0	Newborn screening	3.7	0.1	4.1	4.3	NA	NA	NA	+	–	0	3.7	NA	NA
II	II-1	F	c.565_568del (p.Asp189Metfs*14)	0.5	Obesity, osteoma cutis	0.5	0.3	5.2	16.6	-3.0	2.3	< 2	–	OC, SM, M	0.5	0.5	NA	15.8
III	III-1	F	c.565_568del (p.Asp189Metfs*14)	0	Newborn screening	3.6	-0.8	-0.3	9	-1.4	0.8	–	+	SM, SMt	0	3.6	NA	NA
	III-2	F	c.565_568del (p.Asp189Metfs*14)	14.6	Family screening for PHP	14.6	-1.8	2.3	15.8	-1.9	2.0	> 2	+	Ml	13.5	14.6	NA	15.7
	III-3	M	c.565_568del (p.Asp189Metfs*14)	14.7	Acute abdomen, hypocalcemia	14.7	-0.9	-1.4	18	-2	-0.8	–	+	–	18.4	14.7	NA	–
IV	IV-1	F	c.474T>G (p.Phe158Leu)^b^	6	Routine analysis, elevated TSH	6	NA	NA	22	-3.6	1.9	NA	–	SM	6	6	13,6	22
V	V-1	M	c.91C>T (p.Gln31*)	6	Seizure, hypocalcemia	6	NA	NA	39	-2.6	3.0	NA	–	SM	11	6	NA	–
VI	VI-1	F	c.565_568del (p.Asp189Metfs*14)	12.5	Acute abdomen, hypocalcemia	12.5	0.1	1.2	21	-0.0	1.3	–	–	SM, Ml	12.5	12.5	NA	15.1
VII	VII-1	M	c.85C>T (p.Gln29*)	0	Newborn screening	2	-1.5	1.6	4.5	-0.3	1.7	–	–	OC, SM	0	–	NA	NA
VIII	VIII-1	F	c.1096G>A (p.Ala366Thr)	0.38	Chronic diarrhea	1.3	-1.2	2.3	4.8	-1.4	2.8	<2	+	–	0.4^d^	1.3	NA	NA
Type 1b			*GNAS* locus of methylation alteration																
IX	IX-1	F	NESP55-1, *GNAS*-AS1, *GNAS*XL, *GNAS* A/B	9.5	Routine analysis	9.5	-0.8	0.9	11.5	-0.8	0.9	–	–	–	–	9.5	NA	–
X	X-1	M	*GNAS* A/B	0	Newborn screening	12.3	0.3	1.8	15.7	-0.6	1.0	–	–	–	0	12.3	NA	–
XI	XI-1	M	NESPAS, *GNAS*XL, *GNAS* A/B	11.3	Routine analysis, hypocalcemia	11.3	0.9	-0.5	13.2	1.4	-0.6	–	–	–	13.5t	11.3	NA	–
XII	XII-1	F	NESPAS, *GNAS*XL, *GNAS* A/B	7	Tetany, hypocalcemia	7	0.7	2.0	9.25	0.8	1.9	–	–	SM, SMt	–	7	NA	NA
XIII	XIII-1	F	NESPAS, *GNAS* A/B	11.5	Routine analysis Low fT4	13.4	-0.6	1.7	14.5	0.4	0	–	–	–	11.5t	13.4	NA	–
XIV	XIV-1	M	NESPAS, *GNAS*XL, *GNAS* A/B	11.1	Seizure, hypocalcemia	11.1	-0.8	0.7	29	-1.7	1.1	–	–	SM	11.1	11.1	NA	–
XV	XV-1	F	NESPAS, *GNAS*XL, *GNAS* A/B	12.5	Tetany Hypocalcemia	12.5	-0.3	1.4	19	-0.7	0.9	–	–	–	–	12.5	NA	–
XVI	XVI-1	M	NESPAS, *GNAS*XL, *GNAS* A/B	8.1	Tetany Hypocalcemia	8.1	-0.9	1.2	28	-2.6	2.5	>2	–	SM, MI	11	8.1	NA	–
PPHP																			
XVII	XVII-1	F	NA	5.6	Short stature and osteoma cutis	8	-2.8	-0.8	13.6	-3.1	0.3	–	–	OC, SM	–	–	–	–

AHO, Albright’s Hereditary Osteodystrophy, BMI, Body mass index, F, Female, GHD, growth hormone deficiency, ID, Intellectual disability, M, male, Ml, Madelung deformity, NA, Not available, -, Absent, +, Present, OC, Osteoma cutis, PPHP, Pseudopseudohypoparathyroidism, PTH, Parathyroid hormone, S, Sex, SM, Short metacarpal, SMt, Short metatarsal SF, Skeletal findings, TSH, Thyroid stimulating hormone.

a +Genetic analysis was performed but result could not be obtained.

b Novel mutations.

c Age at diagnosis.

d TRH resistance.

### Anthropometric measurements

At diagnosis, anthropometric data of 8/10 with PHP1A were available; the median height-SDS was -0.9 (-1.8/0.3), and none had short stature. During the final clinical assessment, following a median follow-up period of 4.4 years (0.6-33.0), and at a median age of 16.2 years (4.3-39.0), 9 out of 10 patients with PHP1A had a median height-SDS of -1.9 (-3.6/-0.0), with 44% classified as having short stature. Among the six cases that reached final height (FH), the median height-SDS was -2.4 (-3.6/-0.0), with 66% having short stature (**Table 1**). At diagnosis, the median BMI-SDS of the cases with PHP1A was 1.9 (-1.4/5.2), with four patients (50%) classified as obese. Of the five cases with adequate data on the age at which obesity developed, two (40%) were obese before age 2. At the final clinical visit, the BMI-SDS was 1.9 (-0.8/3.0) (n=9), with 44% of the cases classified as obese (**Table 1**).

The median height-SDS at diagnosis for cases with PHP1B was -0.5 (-0.9/0.9), and none had short stature. Following a median follow-up of 3.8 years (0-19.9), at the final clinic visit, with a median age of 14.6 years (9.3–29), the median height-SDS was -0.7 (-2.6/1.4), with only one having short stature. Among the four cases that reached FH, at a median age of 23.5 years (15.7-29), the median height-SDS was -1.2 (-2.59/-0.6) with only one case classified as short statured (**Table 1**). At diagnosis, the median BMI-SDS of the cases with PHP1B was 1.3 (-0.5/2.0), and following a median follow-up period of 3.8 years (0–19.9) during the final visit, at a median age of 14.6 years (9.3–29), the median BMI-SDS was 1.0 (-0.6/2.5), only one case was obese (**Table 1**).

The case with PPHP was referred at the age of 5.6 years, and she had short stature (height-SDS: -2.8), osteoma cutis, and short metacarpals. Her BMI-SDS was -0.8. 2.4 years later, she was diagnosed with PPHP since she displayed the AHO phenotype without any evidence of hormone resistance. Following 8 years of follow-up, during her final clinical visit, she was still lean; however, her height-SDS further declined (height-SDS: -3.1, BMI-SDS: 0.3) (**Table 1**).

An oral glucose tolerance test was performed in 3 of 10 obese cases with PHP1A, at a median age of 15.8 years (14.5-35). Cases II and III-2 had insulin resistance. Case III-2 was managed with dietary modifications, while metformin was started in case II-1. Case V-1 was diagnosed with metabolic syndrome during adulthood (BMI: 45 kg/m²), and he is currently being treated for type 2 diabetes mellitus and dyslipidemia.

### AHO phenotype

**Figure 2:**
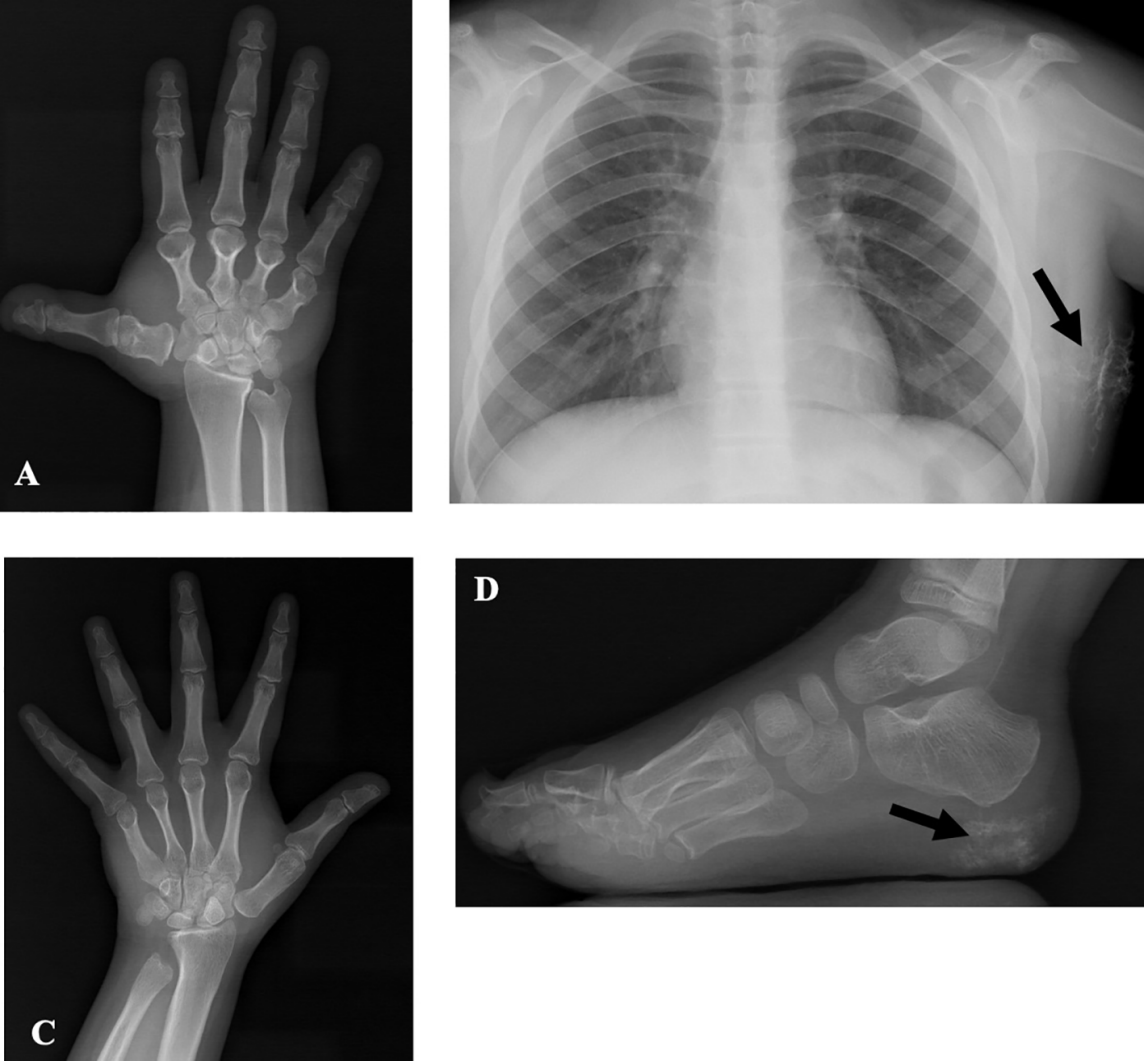
**(A)** Left hand radiograph of case II-1 demonstrating *brachydactyly* and *Madelung deformity*. **(B)** Posteroanterior chest radiography of case II-1, which demonstrates *osteoma cutis* of the left chest wall. **(C)** Left hand radiograph of Case III -2 showing severe *Madelung deformity.* **(D)** Lateral foot radiograph of case VII -1, demonstrating osteoma cutis of the left heel.

Skeletal features of AHO were observed in seven cases (70%) with PHP1A, as well as in the case with PPHP. Only Case II had all AHO features. One patient with PHP1B (Case XVI-1) had initially been clinically diagnosed as PHP1A at age 28 due to the presence of short stature, a round, obese face, short metacarpals, and Madelung deformity. However, he was not short or obese at first presentation (8.1 years old) and following the detection of a *GNAS* methylation defect in genetic analysis, his diagnosis was revised to PHP1B. Osteoma cutis was detected in two cases with PHP1A, as well as the case with PPHP, while it was absent in PHP1B (**Table 1**). One case with PHP1A had a single lesion on the chest, while the other case had multiple lesions on the sole of the foot, the flexor surface of the leg, and the neck (**Figure 2**).

Skeletal findings included isolated short metacarpals (n=3), isolated Madelung deformity (n=1), and short metacarpals with Madelung deformity (n=3). Among cases with PHP1B, three (37.5%) had skeletal abnormalities; two (25%) had short 5^th^ metacarpals, while one (12.5%) had short metacarpals with Madelung deformity. Additionally, one from each group had bilateral short 4^th^ metatarsals. The case with PPHP also presented with short metacarpals (**Figure 2**).

### PTH resistance

PTH resistance was identified in 17 of 19 cases in the cohort. It was detected in 9 PHP1A cases (90%), at a median age of 6 years (0.5–14.7); and in all PHP1B cases (100%) at a median age of 11.2 years (7–13.4) (**Figure 1**). Although the difference in age at PTH diagnosis was not statistically significant among groups (p=0.21), PTH resistance appeared earlier in PHP1A, sometimes in infancy or early childhood, while the earliest age of onset in PHP1B was 7 years. Cases II-1 and VIII-1 were diagnosed at 0.5 and 1.3 years of age, respectively, while they were asymptomatic. One case with PHP1A (Case VII-1; a 4.5-year-old boy) has been followed for congenital hypothyroidism and osteoma cutis (multiple lesions on the sole of the foot, flexor surface of the leg, and neck); however, he has not shown any evidence of PTH resistance according to his last evaluation at the age of 4.5 years. Overall, hypocalcemia was present in 42.1% of those with PTH resistance: 3/9 (33.3%) with PHP1A and 5/8 (62.5%) with PHP1B exhibited hypocalcemia at presentation. Median serum calcium was 7.57 mg/dL (5.54-10.5), and PTH was 431 pg/mL (108.5-1510.7) at the time of diagnosis of PTH resistance. One of the two cases (Case II-1) who manifested PTH resistance at early ages (within the first 2 years of life) had all the features of AHO, while the other (VIII) was only obese.

### TSH/TRH resistance

Hypothyroidism was identified in 15/19 cases (79%) of the PHP cohort: all ten cases (100%) with PHP1A and 5 cases (62%) with PHP1B. Four patients (three PHP1A, one PHP1B) were identified through newborn screening. The age of detection for hypothyroidism, excluding cases identified through newborn screening, was comparable in PHP1A and PHP1B, with median ages of 11 years (0.4–18.4) and 11.3 years (11.0-13.5), respectively (p=0.20) (**Figure 1**). Median TSH was 8.5 μIU/mL (2.6–20.9) (normal range: 0.4-5.3), and median FT4 was 11.6 pmol/L (5.3-15.8) (normal range: 7.8-13.7). In seven cases of PHP, hypothyroidism preceded PTH resistance, representing 50% of PHP1A cases and 25% of PHP1B cases.

In addition, during initial hormonal work-up, TSH was <4.5 μIU/mL and FT4 was low in three cases (one with PHP1A [Case VIII-1; FT4: 4.86 pmol/L, TSH: 1.94mIU/L]; two with PHP1B [Case XI-1; FT4: 7.8 pmol/L, TSH: 3.5 mIU/L and XIII-1; FT4:8.2 pmol/L, TSH: 2.64 mIU/L]), leading to a diagnosis of central hypothyroidism. Following Na-levothyroxine treatment, FT4 was normalized, and TSH levels were suppressed below the lower range, supporting the diagnosis of central hypothyroidism. Hence, the remaining pituitary hormones were also evaluated, and deficiencies in all three cases were excluded. Hypophyseal magnetic resonance imaging was performed in case XIII-1, which was normal.

### Gonadotropin resistance

Gonadotropins were measured in 14/19 cases (4 girls, 2 boys with PHP1A; 3 girls, 4 boys with PHP1B; 1 girl with PPHP). Gonadal dysfunction was clinically identified in 4 of 6 patients (66%) with PHP1A, all of whom were female. In these 4 cases, FSH was at the upper limit of laboratory norm for age and sex, despite normal levels of estradiol. Clinical and laboratory findings of cases with and without gonadal dysfunction are presented in **Table 2**. Gonadotropin resistance was not detected in boys. Among males, one was married and had a child; the remaining individuals were single and childless.

**Table 2:** Diagnostic findings, gonadotropin and sex steroid measurements of four females with gonadotropin resistance and pubertal cases with normal gonadal function.

Cases	Age (years) Symptoms	Finding	LH (mIU/mL) (1.8-11.78)	FSH (mIU/mL) (3.03-8.08)	Estradiol (pg/mL) (21–251)	Tesetosterone (ng/dL) (151-794)
Gonadotropin resistance						
II-1	16.6	Oligomenorrhea	9.42	9.3	41	12.5
III-2	15.8	Oligomenorrhea	2.34	8.96	15	–
IV-1	22	Infertility	–	–	–	–
VI-1	21	Amenorrhea	12.1	8.65	33	–
Normal gonadal function						
Males (n=6) (median, min-max)	18 (13.2-39)	–	3.87 (1.89-7.6)	3.68 (1.74-7.57)	–	510.9 (318.34-610.31)
Females (n=4) (median, min-max)	13.6 (13.4-19)	–	3.36 (0.39-4.72)	5.58 (5.08-6.06)	89 (13-124)	–

LH, luteinizing hormone; FSH, follicule-stimulating hormone; -, absent.

### Growth hormone-releasing hormone resistance

GH stimulation tests were performed only in one case (Case IV-1) among PHP1A patients, and the GH response were low (peak growth hormone response were 3.6 and 5.2 ng/mL). She was lost to follow-up until age 22, when her FH SDS was -3.58. GH stimulation tests were also performed in the case with PPHP, and the peak GH response (9.41 ng/mL) was within normal limits.

### Neurocognitive findings

Intellectual disability was observed in 5/10 (50%) of cases with PHP1A; two exhibited developmental delay, while three displayed mild cognitive impairment on WISC-R. The remaining 5 cases with PHP1A had normal academic achievement. Psychometric tests were not performed in cases with PHP1B, since intellectual disability was not suspected during clinical evaluation, and they had normal academic performance and achievement, some pursuing careers as physicians and lawyers.

### Phenotypic variability of cases carrying the same *GNAS* variant

Three siblings with PHP1A from Family III, their mother, and Case VI-1, as shown in **Table 1**, harbored an identical genetic variant. The timing and type of hormone resistance, as well as AHO features, were quite heterogeneous. The mother of Family III only had short stature. Hormone resistance and other AHO features were not detected, suggesting that the mother inherited the mutant *GNAS* allele paternally.

## Discussion

In the current study, thorough clinical and endocrine follow-up of 19 cases from 17 families was presented to identify the natural course of each PHP type. All PHP1A and PHP1B cases were diagnosed with PHP once PTH resistance was identified, except for those one whose diagnosis was established with hypothyroidism and osteoma cutis. One case was diagnosed with PPHP based on AHO features in the absence of hormone resistance. Overall, PTH resistance was the most frequent endocrine finding. Although 38.8% of cases presented with TSH resistance, PHP was not suspected, resulting in a diagnostic delay of up to 12 years. In rare instances, osteoma cutis or obesity were the initial findings.

At diagnosis, none of the PHP1A cases had short stature, but 44% developed short stature (44%) by the last clinic visit; 66.6% was short at FH. In the literature, nearly one-third of cases with PHP1A and PPHP were reported to have short stature at diagnosis (23). de Sanctis et al. reported that 3/10 cases with PHP1A had short stature, two of whom were diagnosed with GH deficiency. The other one, whose height-SDS was -3.5, had normal GH response during GH stimulation test (24). Mantovani et al. observed that GH treatment improved growth velocity and height outcome in eight PHP1A with GH deficiency (25). In the current cohort, Case IV-1 with severe short stature (FH: -3.5 SDS) was diagnosed with GH deficiency; however, GH treatment could not be initiated, and she was lost to follow-up. The impact of GH treatment on FH in patients with severe growth retardation in PHP remains to be determined. Despite a satisfactory GH response in the GH stimulation tests, the FH in our case with PHPP was -3 SDS. Thus, resistance to GHRH is unlikely to be the only driver of short stature in PHP1A cases. Gsα haploinsufficiency in the growth plate likely contributes to the short stature in patients with PHP1A and PPHP, as described previously in mouse models (26). Only one case with PHP1B had a short FH.

Patients with PHP1A and PHP1B exhibit food-seeking behavior or hyperphagia from an early age in comparison to those with exogenous obesity (27). In the current study, the prevalence of obesity in PHP1A was 50%. Obesity is known to be less common and less severe in PHP1B (28). Likewise, our cases with PHP1B were not obese at the time of diagnosis, and only one case developed obesity during the final clinic visit. However, a recent study indicated that the prevalence of obesity was higher in both PHP1A and PHP1B, with 94% of PHP1A patients and 50% of PHP1B patients classified as obese (27). Despite consensus reports and previous studies noting a higher risk of obesity before the age of two in PHP1A (7, 28), in the current study, only 20% developed obesity before the age of two.

Oral glucose tolerance tests revealed insulin resistance in two, and type 2 diabetes mellitus and dyslipidemia in one case with PHP1A with severe obesity during adulthood. This aligns with previous evidence indicating reduced insulin sensitivity and an increased risk of type 2 diabetes in adults with PHP1A (29). These findings highlight the importance of long-term metabolic surveillance in obese PHP1A patients.

PTH resistance tended to occur earlier in cases with PHP1A than in those with PHP1B (6 vs 11.2 years; p=0.21), although the difference did not reach statistical significance. Cases II-1 and VIII-1 were diagnosed before the age of two. Previous studies indicate that PTH resistance in PHP1A is generally absent at birth. It is typically identified between the ages of 4 and 6, suggesting that it may develop from infancy through the second decade of life (9). Similar to our findings, an increase in PTH has been reported as the initial sign of PTH resistance in PHP1As. Although calcium levels were normal, 80% already had elevated PTH levels, which helped enable earlier diagnosis (30). The results indicate that serum calcium and PTH levels should be monitored regularly in cases of PHP1A, with intervals of 3–6 months during the first few years of life, transitioning to annual assessments, and more frequent follow-ups during periods of rapid growth (31). PTH resistance developed earlier (6 months of age) in our single case with a complete AHO phenotype (Case II-1). The association between the complete AHO phenotype and the earlier onset of hormone resistance has not been previously evaluated. PHP1B cases developed PTH resistance during later childhood or adolescence. The youngest identified case of PHP1B with PTH resistance was 7 years old, suggesting that annual monitoring starting from school age may be reasonable (3).

TSH resistance was the second most common endocrine finding identified in 78.9% of the overall cohort. This is comparable to previous reports (30). PHP is often identified with PTH resistance. Typically, other hormone resistances are evaluated after the diagnosis of PHP, which supports the expectation that TSH resistance will be identified concurrently or shortly after PTH resistance. Jiang et al. (30) noted that PTH and TSH resistance in PHP1A cases were diagnosed at similar ages (5.4 vs. 5.5 years). However, we found in the current study that TSH resistance preceded PTH resistance in 38.8% of cases. Some of these were diagnosed through newborn screening. The prevalence of hypothyroidism identified through the newborn screening program was 30% in PHP1A and 12.5% in PHP1B. There are only a limited number of case reports of PHP1Bs initially diagnosed through newborn screening for congenital hypothyroidism (32).

In our cohort, two cases with PHP1B and one with PHP1A had low FT4 without elevated TSH. Following Na-levothyroxine treatment, FT4 was normalized, and TSH levels were suppressed below the lower range, further supporting the diagnosis of central hypothyroidism. Hypophyseal magnetic resonance imaging was normal in one of these cases. When these findings are considered, it may be proposed that these PHP1A and PHP1B cases had impaired thyrotropin-releasing hormone (TRH) action. TRH is known to exert its effects through G protein-coupled TRH receptors (TRH-R), which signal predominantly through G_q/11._ In addition, under certain circumstances, TRH-R was shown to activate multiple second messenger pathways through interactions with Gsα and the alpha-subunit of the inhibitory G protein (33). Prolactin release is also driven by TRH-R, which is functionally linked to Gsα in lactotrophs (34). To determine the underlying mechanism of hypothyroidism, Balavoine et al. (35) performed a TRH stimulation test in ten patients with PHP1A. In more than half of patients, the TRH stimulation test showed blunted responses of fT3, fT4, and TSH. A reduced prolactin response to exogenous TRH was also reported. Previous literature indicates that attenuated responses were observed in 22 of 32 patients with PHP1A who underwent the TRH stimulation test, suggesting that TRH resistance may be part of the spectrum of hormone resistance in PHP (35). Consequently, PHP must be included in the differential diagnosis when isolated central hypothyroidism occurs in the absence of other pituitary hormone deficiencies. Notably, we detected evidence of TRH resistance not only in one of the PHP1A cases but also in two PHP1B cases. As in resistance to other hormones, predominantly maternal Gsα expression in TRH-responsive tissues may contribute to the development of TRH resistance in these patients, in whom genetic or epigenetic *GNAS* alterations affect the maternal allele. In addition, given the incomplete penetrance of TRH resistance, the degree of allelic bias in Gsα expression likely varies significantly among individuals.

In the current study, despite normal gonadotropin levels (except for an FSH at the upper limit of normal), 21% had clinical evidence of gonadal dysfunction (including menstrual irregularities and infertility), suggesting gonadotropin resistance. All were girls and were diagnosed with PHP1A. Gonadotropin resistance has been reported in 8.6% of cases with PHP1A and 3.5% of cases with PHP1B (36). This discrepancy can be attributed to varying definitions of gonadotropin resistance and to our focus on its clinical manifestations. In the current study, gonadotropin resistance was not observed in boys. Previous studies have reported cryptorchidism and hypogonadism in boys with PHP1A (3). Central precocious puberty has also been documented in two boys with PHP1A (37, 38). Our findings suggest variability in the severity and timing of gonadal involvement, which requires close monitoring of gonadal function. Additionally, patients and their families should be counseled regarding the risk of gonadal dysfunction and reproductive issues, even in the absence of laboratory abnormalities.

If AHO features are recognized in a child, an assessment for PHP1A should be conducted, as previously highlighted (3, 7). In the present study, 70% of cases with PHP1A displayed AHO features; however, a complete AHO phenotype was observed only in Case II-1. Among PHP1A cases with AHO, 50% had intellectual disability, suggesting that it may not be a cardinal finding. An incomplete AHO phenotype may also be seen in cases with PHP1B, usually leading to a misdiagnosis of PHP1A (16). Indeed, two components of the AHO phenotype were present in Case XV1-1, and he was initially followed for PHP1A until genetic analysis confirmed PHP1B. Therefore, genetic analysis is fundamental to confirm the PHP subtype, particularly in individuals with an incomplete AHO phenotype.

Four cases (Family III, n=3 and Family VI, n=1) harbored the same genetic variant; however, their clinical manifestations were heterogeneous. Evaluating *GNAS* genotype-phenotype correlations, Cipriano et al. recently showed that the frequencies of short metacarpals, a round face, and intellectual disability in individuals carrying the same variant were 74%, 71%, and 69%, respectively (37). Not only the clinical findings, but also the age and severity of hormone resistance were heterogeneous among our patients carrying the same variant. Studies have previously pointed out that identical mutations may lead to divergent clinical outcomes without a clear genotype-phenotype correlation. Epigenetic factors, including environmental and genetic modifiers may contribute to variations of phenotype. However, clinical findings, age of onset, and severity varied even within the same family, where genetic and environmental characteristics are expected to be similar. Therefore, additional factors may alter presentation and disease course.

Several limitations should be pointed out. Firstly, genetic analysis could not be performed in one case, and results could not be obtained in another case. These were classified and evaluated based on clinical findings, which may have affected our results. Secondly, it is important to acknowledge the constraints inherent to a retrospective study spanning nearly three decades, including the lack of significant clinical, laboratory, and follow-up data, such as diagnostic thyroid function tests, gonadotropins, or age of obesity onset. Thirdly, it is important to emphasize the small sample size due to the rarity of PHP, as well as variations in laboratory assays and diagnostic methodologies over time.

## Conclusion

Our findings highlight the clinical heterogeneity of PHP, wherein identical genetic defects can result in varying phenotypic features, including differences in age of onset and severity of hormone resistance. Moreover, similar clinical manifestations can arise from different genetic variants. Therefore, early molecular diagnosis is essential for individualized follow-up. The prevalence and severity of growth impairment increase with age. Obesity is a significant finding; however, its onset does not invariably occur during infancy. An incomplete AHO phenotype is not unique to PHP1A and may be observed in PHP1B, whereas a complete AHO phenotype is relatively specific to PHP1A. PTH resistance remains the defining diagnostic characteristic of PHP, even if TSH resistance can be detected as early as the neonatal period. Importantly, TRH resistance may be the underlying etiology of central hypothyroidism or low FT4 with inappropriately normal or low TSH levels seen both in PHP1A and PHP1B. Despite normal gonadotropins, clinical findings of impaired gonadal function indicative of gonadotropin resistance may be observed.

## Data Availability

The original contributions presented in the study are included in the article/supplementary material, further inquiries can be directed to the corresponding author/s.
